# Wildfire mobilization of arsenic into water resources: A scoping review of current literature

**DOI:** 10.1371/journal.pwat.0000549

**Published:** 2026-08-19

**Authors:** Jordan Willeford, Leigh Moorhead, Kai Malone, Alexis Payton, Janice S. Lee, Julia E. Rager, Stephen D. LeDuc

**Affiliations:** 1 Department of Environmental Sciences and Engineering, University of North Carolina at Chapel Hill, Chapel Hill, North Carolina, United States of America,; 2 United States Environmental Protection Agency, Durham, North Carolina, United States of America

## Abstract

Increasing wildfire activity poses a threat to water resources via the mobilization of hazardous materials such as arsenic (As). While As contamination of water resources is well studied, the fate and transport of As impacted by wildfire are not well characterized. This literature review synthesizes studies addressing the questions of: (1) Does fuel type influence how As is mobilized by wildfire?; (2) What mobilization pathways are most common for As when impacted by wildfires?; and (3) How are surface and groundwater As concentrations impacted by wildfire mobilization of As? To address this goal, articles were queried using Boolean search strings in Google Scholar, PubMed, and Web of Science and screened for relevance using PECO (Population, Exposure, Comparator, and Outcome) criteria. Twenty articles met the criteria, showing wildfire mobilization of As and changes in concentrations or loading of As to surface or groundwater. The majority of studies (16 of 20) showed an increase or a likely increase in As in waterbodies post-wildfire. Runoff to surface water was by far the dominant pathway. Average values typically did not exceed drinking water standards, although six studies reported instances where this occurred, with one reporting exceedances in treated drinking water itself. The largest value of dissolved As reported across surveyed literature (70 μg/L) was measured downgradient of a legacy mining operation post-wildfire. Higher values were also observed downstream of burned agricultural or urban areas, illustrating how human activities concentrate As, which can then be subsequently mobilized by wildfire. In contrast to surface water, groundwater impacts were inconsistent in the limited literature, with one study showing an increase in post-wildfire As, while the other two studies showed no-change. Overall, these results suggest communities may need to test drinking water supplies for As following wildfire, especially in cases where mining, agricultural, or urbanized areas are burned.

## Introduction

Wildfire activity is prevalent in many areas around the globe, and can pose a threat to both the environment and human health [1]. In the United States (US) alone, almost 700 thousand wildfires burned over 32 million hectares between 2014 and 2024 greatly exceeding that in previous decades (e.g., nearly doubling the land area burned between during the 10-year period of 1990 and 2000) [2]. In addition to the direct damage of wildfire events, wildfires can also cause downgradient environmental consequences. For example, wildfires can mobilize metals and other chemicals into waterbodies causing further impacts [3,4]. These potential secondary effects of wildfires could have long-term effects on the environment and human health.

Amongst metals, there is concern that wildfires can mobilize the highly toxic metalloid, arsenic (As), into waterbodies, and ultimately contaminate drinking water sources [5]. Arsenic is a naturally occurring element, occurring in both organic and inorganic forms, and often found in the earth’s crust, soil, and rock formations, with the potential to impact human health [6,7]. Organic As contains hydrogen and carbon atoms, whereas inorganic As is combined with sulfur, chlorine, or oxygen [8]. In general, exposure to organic As compounds is considered less toxic than inorganic As compounds [9]. Organic As compounds include arsenobetaine and arsenocholine found in fish and shellfish, as well as herbicides disodium methylarsenate (DSMA) and monosodium methylarsenate (MSMA) [9]. The most common forms of inorganic As are arsenate (As(V)) and arsenite (As(III)) [9]. Arsenic can bind to form compounds and undergo various reactions, impacting both its mobility and toxicity [9]. Lifetime or chronic exposure to As is a concern due to chronic health effects associated with repeat exposure conditions [6,10]. Inorganic As is a known human carcinogen, and it is associated with various non-cancer health outcomes including diabetes, circulatory diseases, pregnancy complications, nonmalignant circulatory diseases, skin lesions, and neurodevelopmental and fetal development/birth complications [11–13].

Throughout the US, there are areas with both naturally high As concentrations and high wildfire hazard potential [14–17]. Arsenic may mobilize post-wildfire into surface water resources through runoff and flooding, and through direct deposition in smoke and ash impacting critical water resources used for human consumption [5, 18]. Arsenic can bioaccumulate in vegetation and often binds to soil materials, which when burned increase mobility [18–20]. Groundwater resources may also be impacted by As from wildfire and subsequent leaching [18,21]. Treated surface and groundwater used for municipal drinking water are regulated in the US by the Clean Water Act and Safe Drinking Water Act, which set acceptable levels and requirements for mitigation and filtration to prevent potential exposures of As to humans. The national primary drinking standard for water resources, both in surface and groundwater, is 10 μg/L in the US [6]. The Clean Water Act also provides ecological protection to aquatic environments of the nation’s water resources, putting in place criteria to protect waterways to reduce and prevent pollution [22,23]. By contrast, however, private well water sources are not regulated under the Safe Drinking Water Act and therefore populations relying on them may be more vulnerable [22].

Given the increase in wildfire activity, including in areas impacted by As contamination, it is critical to understand the potential for contamination post-wildfire. While prior reviews have synthesized wildfire impacts on water quality more broadly (e.g., Paul et al. 2022 [24] and Smith et al. 2011 [25]), the present study focuses specifically on As, aiming to complement the existing literature by providing greater detail on a specific contaminant of concern. Specifically, this review fills an important knowledge gap on As mobilization in water sources due to wildfire events.

## Materials and methods

### Overview of literature review steps

The aim of this literature review was to synthesize current published evidence, identify potential research gaps, and highlight areas for future research surrounding wildfire-associated mobilization of As in the environment. Notably, this review could not be implemented as a classical systematic review, because criteria for study quality and risk of bias evaluations for the types of studies included in this review do not currently exist. However, methods implemented in this literature review follow those of prior studies, namely by defining a clear set of research questions, developing a conceptual model, choosing appropriate literature databases, and extracting information on the directionality, magnitude, and duration of impacts [26]. Similar to previously published evidence maps, Population, Exposure, Comparator, and Outcome (PECO) criteria were developed to help find and assess relevance of literature [27]. This review was conducted following these steps: (1) generation of a conceptual model; (2) development of research questions; (3) development of PECO criteria screening; (4) search string development; (5) study eligibility and inclusion/exclusion criteria development; and (6) literature screening that incorporated findings from steps 1–5, which included a pre-screening step reviewing titles/abstracts, followed by full text review.

This literature review first generated a conceptual model, a visual representation of research questions and guidance for research [28,29]. The structure of the conceptual model used to organize this review followed a similar structure to an article focusing on antibiotic resistance in a non-built environment [28]. The structure in this study began with source, then how it spread, finally ending with fate and transport. The conceptual model for this review consisted of media impacted directly by wildfires, then potential mobilization pathways that As may undergo, and impact on water resources based upon an initial review of existing literature. Upon completion of the conceptual model, the following research questions were developed: (1) Does fuel type influence how As is mobilized?; (2) What mobilization pathways are most common for As when impacted by wildfires?; and (3) How are surface and groundwater As concentrations impacted by wildfire mobilization of As? PECO screening criteria were then developed, as further described below. From these findings, search terms were developed in three different databases: Google Scholar (Google Scholar [Internet]. Available from: https://scholar.google.com/: **6/13/2024**); PubMed (PubMed [Internet]. PubMed. Available from: https://pubmed.ncbi.nlm.nih.gov/: **6/14/2024**); and Web of Science (Clarivate [Internet]. Available from: https://clarivate.com/academia-government/scientific-and-academic-research/research-discovery-and-referencing/web-of-science/: **6/13/2024**). Lastly, articles were identified and organized into categories of relevant information and evaluated for inclusion/exclusion criteria based on the research questions and PECO relevance screening, followed by full text screening. These steps are further detailed below.

### Literature search and pre-screening

This study’s literature review used keywords and Boolean search strings to identify and aggregate relevant literature. The initial search was conducted using Google Scholar, while a parallel search was conducted by HERO (Health and Environmental Research Online) staff in PubMed and Web of Science. The US Environmental Protection Agency maintains HERO, a peer-reviewed literature database and service, to assist with development of reports and research. The initial Google Scholar search was conducted using an optimized search string to collect literature based on the PECO criteria. Next, a parallel search completed by HERO ensured the search was fully optimized and would not miss relevant articles. The literature search time frame of the initial Google Scholar and HERO searches were completed by 6/13/2024 and 6/14/2024, without restrictions on the publishing date of literature included. Supplementary targeted searches were conducted if the primary literature search did not capture certain environmental stressors, media, and modes of mobilization of high relevance to the outlined research questions. The three different searches were conducted in tandem to ensure that all relevant literature from each of the databases based on the PECO criteria would be included.

The search strings developed for this review were created to capture literature based on the PECO criteria. Keywords used in the search strings fell into the three categories of (1) mobilization pathway, (2) fuel type and impacted media, and (3) impact on water resources, mirroring the PECO criteria. These keywords were drafted in the search string to ensure at least one keyword was included in each of these categories. To capture all articles relevant to answering the research questions, parallel and targeted searches were built upon the search strings used in the primary searches. The secondary search was optimized with the help of HERO staff to ensure relevance of search terms. Targeted searches removed irrelevant terms to specific mobilization pathways and inserted synonyms or related terms to capture literature not captured in the broader searches. The additional pathways targeted were landslides, fire mitigation and control, private wells, groundwater, and leaching. The Boolean search string and associated terms can be found in S1 Text in Supplemental Materials. The articles internally aggregated and identified by HERO were reviewed and analyzed to ensure consistency in the analysis and findings. The literature yielded through Google Scholar and HERO search were reviewed for relevance to each of the research questions and factors being assessed. Articles were initially pre-screened based on the relevance of the title and abstract to the research questions. Upon completion of the pre-screening of the title and abstract, the articles included underwent further screening applying PECO criteria.

### PECO relevance and full text screening

The PECO relevance criteria were applied following the title and abstract pre-screening using a developed PECO statement. The PECO statement for this review was, “In areas impacted by wildfires, As concentration in water is influenced by media type (soil, ash, smoke, and vegetation), mobilization pathway (leaching, runoff, and direct deposition), and time elapsed post-wildfire.” This PECO statement addresses all aspects of the aim of this review, and is described in Table 1. The PECO and study types of literature included were visualized through an evidence map. Literature tagged as supplemental had valuable insights or added context that may not have been captured in the PECO-relevant literature. Articles tagged as supplemental included review articles, meta-analysis articles and articles containing some aspects of the PECO criteria but not all. The final analysis of articles included full text screening involving the manual reading of each article and a typed summary of key details, including relevance to the literature review, results, and conclusions. All PECO-relevant literature was extracted for directionality, magnitude, and duration of impacts, following the methods of Paul et al. [24], and summarized by mobilization pathway, fuel type, and impacted media.

## Results and discussion

### Overall literature screening results

#### Quantified results of literature searches.

The primary screening of literature across Google Scholar, HERO, and targeted parallel searches yielded 676 citations, after removing duplicates, broken links, and otherwise incomplete citations (S1 Table in Supplemental Tables). These articles underwent title and abstract screening. Following this initial screening, 105 articles received additional full-text screening for relevancy to the PECO criteria (see below). The literature search process from the primary and parallel searches is outlined in the Prisma diagram (Fig 1).

#### PECO criteria results.

The PECO criteria were applied to each of the pre-screened 105 articles to assess relevance to the PECO parameters outlined in the methodology in Table 1. The final review of the articles yielded 20 PECO-relevant articles and 56 supplemental articles. The list of included articles identified as PECO-relevant are available in S2 Text in Supplemental Materials, and the list of articles included as supplemental are available in S3 Text in Supplemental Materials. The literature tagging tree highlights studies identified that met PECO criteria (Fig 2). Additionally, the directionality, if applicable, and mobilization pathway discussed in the literature are shown in the evidence map.

The most common measured outcome was surface water As concentration rather than groundwater As concentration. The exposure for all articles was the same, which was wildfire exposure. The most common comparators were either pre-wildfire As concentration in water resources or focus and comparison of specific impacted media (such as soil, vegetation, man-made structures, and building materials). The most common locations of the literature were Canada, Portugal and the western United States.

Additional details of the results of the review are shown in Fig 3 and Fig 4. Fig 3 describes the frequency of PECO criteria within the literature included. The population serves as a proxy for the individual articles which are used as the basis of comparison between articles showing differences in the influence of burned media and whether groundwater or surface water are impacted post-wildfire. Fig 4 indicates the directionality and percent change of surface and groundwater As concentrations. The water resources in this figure are both finished and unfinished water (i.e., treated drinking water and untreated water). Code used to generate Fig 3 and Fig 4 is available on Github [30].

### Literature synthesis by mobilization pathway

#### Runoff as a mobilization pathway for as.

In the literature found in this review most reported runoff as the mobilization pathway for As into water resources after wildfire. The mobilization pathway was not always clear; however, over half of the studies (12 out of 20 papers) exhibited runoff as the main pathway or runoff was at least a likely pathway (S2 Table in Supplemental Materials). All of these studies reported runoff to surface water bodies showed an increase in As concentrations as a result. Wildfires increase runoff potential due to several key factors, including: the mortality of vegetation, decreasing canopy interception and plant uptake of precipitation, increased erosion due to the lack of vegetation, and decreased soil absorption after heating [6,21]. The lack of barriers and changes to soil following wildfire increases the likelihood of overland flow and erosion of materials from topsoil carried into nearby surface water [21]. Notably, many of the studies recorded observations of increased As in stormwater or associated with precipitation events post-fire (e.g., Burton et al 2016 [18]).

Maximum As concentrations in surface water from post-wildfire runoff exceeded 10 μg/L in six of the 12 studies, with almost all of these in untreated water. The highest concentrations following wildfire noted across the studies (70 μg/L dissolved As; 740 μg/L of total As) were measured downstream from a legacy mining operation, coinciding with storm events [21]. Arsenic concentrations also were higher in streams draining areas where buildings were burned versus wildland burning [18]. Both cases suggest human activities can concentrate As on the landscape, resulting in higher concentrations when mobilized by runoff post-wildfire. Most studies of As concentrations from runoff did not measure As more than a year following wildfire, but a couple of studies noted higher concentrations after five and even 10 years post-wildfire—the latter in treated drinking water [5,21]. Taken as a whole, the literature suggests As concentrations are often highest immediately following the first few storm events after wildfire, yet can remain elevated for multiple years.

Both the concentration of As on the landscape by human activity and the duration of effects fit within the broader literature on metals mobilized by wildfire. For instance, Boaggio et al. reported higher values of lead, nickel, copper, and other metals, in addition to As, in air samples downwind of wildfires destroying houses, vehicles, and other anthropogenic materials in California [31]. Likewise, these metals were reported in water draining burned urban areas [32]. Moreover, our finding here that As may be elevated for multiple years post-wildfire is consistent with that of metals generally post-wildfire. For example, consistent with this finding, Paul et al. found metals tended to be elevated in waterbodies the first year following wildfires burning both anthropogenic and natural fuels, but could last up to five years afterwards, with even greater duration for mercury [24]. Thus, it is likely that metals, including As, can be a stressor to waterbodies for multiple years following a wildfire.

#### Direct deposition as a mobilization pathway for as.

The next most common mobilization pathway discussed in the literature is direct deposition of ash and smoke into surface water resources (ash and smoke were not discussed in the literature in the context of groundwater resources). Of these studies, three exhibited pathways that were deposition or simulated deposition (as in the case of lichen ash being added to water in Manasypov et al. 2017 [18–20]); one was likely deposition; and one more had a possibility of deposition, although the likeliest pathway was runoff. All of these studies resulted or likely resulted in increases in As concentrations in waterbodies. As previously mentioned, ash poses a risk of increasing As concentration in soil and in runoff. However, another mobilization pathway is through the direct burning of fuels containing As, such as soil, vegetation, building materials, etc. [18–20]. Ash and smoke can then be carried by wind to nearby surface water resources [33]. The supplemental literature indicates that As emissions from wildfires can be globally significant, especially when mining landscapes burn [34]. For instance, four wildfires in the mining region around Yellowknife, Canada in 2023 resulted in an estimated 2–9% of total global As emissions from all natural sources globally [34]. Moreover, As associated with fine particulate matter (PM_2.5_) in smoke can be carried hundreds of kilometers downwind of wildfires [31]. This study found that As in PM_2.5_ was associated with specific, destructive wildfires, such as the Camp Fire in 2018 which burned portions of the town of Paradise, California. In this case, As was measured in air samples at a ground-level monitor near Modesto, California, over 200 kilometers from the fire epicenter [31].

Of the three studies with deposition as the major pathway, only one showed As concentration greater than 7 μg/L, a level equivalent to Australian drinking water standards, where the study was conducted [35]. Another study showed As increased in sediments associated with depositional layers of charcoal in lakes, a metric not directly comparable to drinking water standards. Moreover, the duration of impacts was not addressed either. It may be that As concentrations do not reach the magnitude of those from runoff and are shorter-lived given that the source of fine particulates and ash may be exhausted more quickly than sources of runoff. Indeed, recent literature on nutrients in smoke and their downwind impacts suggest these pulses may be short-lived. Air quality samples of nutrients quickly return to baseline levels once the smoke from a wildfire clears, and algal blooms in downwind lakes potentially caused by additions of these nutrients appear to be relatively ephemeral as well [36,37].

#### Leaching as a mobilization pathway for as.

There was only one PECO-relevant article with leaching as a mobilization pathway, three with likely leaching, and one more with the possibility for leaching, although runoff was the likely dominant pathway. Of the three articles with leaching or likely leaching, only one showed an increase in As concentrations, and runoff may have had pronounced effect in this particular study [5]. Based on supplemental literature (such as review articles, meta-analysis, and articles meeting part of the PECO criteria), however, there is some evidence to suggest that wildfires may influence leaching and As soil interactions. In a supplemental study examining the composition of ash and soil in orchards in California, it was found that the ash is incorporated into the soil and undergoes partial dissolution [33]. Additionally, it was found that under post-wildfire conditions, water extractable As concentration in ash exceeded 5 mg/L, the criteria concentration, indicating leaching potential [38]. When leaching was assessed directly in a study examining the impact of wildfire on soil properties in Euboea, Greece, the leaching potential of As was highly dependent upon the presence and the subsequent pyrolysis of organic matter [39]. Under these conditions, As is more likely to undergo speciation from As(V) to As(III), which is a more mobile and toxic form of As. Additionally, As leachability was found to likely increase when the soil was more acidic [39].

### Literature synthesis across the second category: Impacted media

#### Soil as a media type impacted by wildfire.

Beyond the mobilization pathway, the As concentration in water is impacted by the media affected by wildfires, including soil, vegetation, and anthropogenic material. The literature that discussed soil largely focused on how soil erodes and carries bound and mixed materials in runoff. Soil type and mineralogical composition affect As mobilization potential due to wildfires because As’s propensity to bind to soil is dependent upon soil chemistry. Supplemental literature indicates that As in the soil can bind to materials in the soil, such as iron, due to electron transfer [40,41]. Arsenic can be mixed into the soil through a few different methods. Pre-wildfire As can be deposited in soil through naturally occurring processes (e.g., wet deposition) and can be deposited from industrial processes through erosion from industrial sites [42,43]. Post-wildfire As can also be mobilized through runoff from drainage areas in mines and industrial sites, as well as the deposition of As-containing ash into the soil [20,21].

The influence of wildfire temperature on the mobility of As in the soil was considered in the supplemental literature, like Sutton et al. (2024) [34]. In wetland soils, As was more likely to be emitted into the atmosphere from more intense, high severity wildfires, whereas in unburned areas or low-temperature smoldering wildfires, As was more likely to be eroded [34]. In laboratory experiments, heating various types of soil indicated that As was most likely to become a more mobile species (As(III)) at 300–400 degrees Celsius. At these intermediate temperatures, As partially reduces from As(V) to As(III), which is more mobile. At the hottest temperatures (800 degrees Celsius), As re-oxidizes back to As(V). At lower temperatures, As did not reduce as efficiently. Soil organic matter content is linked to increased speciation of As, and once the organic matter ceases burning or is completely consumed, As remains or returns to As(V) [39,44]. The solubility of As increases with heating, which leads to decreased levels of As in the soil. When heated, materials release As-bound compounds in soil [45].

#### Vegetation as a media type impacted by wildfire.

PECO-relevant literature suggests that As bioaccumulates in vegetation from soil rich in As [20]. When this vegetation is burned, As within the vegetation is released through smoke and ash [20]. Supplemental and PECO literature both indicate the amount of As in ash and in surface water post-wildfire can be related to the completeness of vegetation burned (burn severity) [3,46]. Supplemental literature supports that the burning of vegetation can emit As into the atmosphere [39], and As in ash can be eroded and carried both into surface water and mixed into soil [46]. Another factor that was discussed in the literature was the variation based on type and parts of the vegetation burned. Liu et al., (2023) found that the concentration of As released in smoke from five plants ranged from undetectable levels to ~67 μg/m^3^, with camphor leaves (~26 μg/m^3^) and branches (~67 μg/m^3^) contributing the most to the As concentration in smoke produced by the five plants [47]. By contrast, in ash, the largest concentrations of As were produced by Ramie, Miscanthus sinensis, and camphor branches, which contributed 33%, 32%, and 24%, respectively to the total As content in residual ash [47]. Arsenic concentrations in the residual ash samples were primarily composed of environmentally mobile and bioavailable species, like soluble and reducible acids, which can readily leach into water and absorb into plants [39]. While As concentrations in smoke and ash vary greatly across vegetation types and burn conditions, the literature supports that As emissions from burning vegetation are a relevant source in the mobilization of As by wildfire.

#### Land-use as a media type impacted by wildfire.

Land-use can also affect As mobilization by wildfire. Mining often introduces contaminants into the environment due to drainage and processes required to extract metals and minerals. One PECO-relevant article discusses the impact of mining on As levels in surface water and the relationship between the presence of mining waste during wildfires [21]. When mining is performed, underground materials are loosened, released, and exposed, making them prone to remobilization through runoff and erosion. The increase in exposed As (among other materials) leads to greater potential for runoff through mine drainage as well as direct removal from mining surrounding targeted materials [21]. The literature indicates that the exposure of targeted materials in mining and their disturbance/mobilization leads to them depositing in surface soil, becoming mixed with other materials within the soil, and being directly washed out into surface water. Literature indicates that when mining is present, the As concentration increases in soil and surface water sharply after wildfires impact the area [21]. Under wildfire conditions, As is mobilized from soil, drainage from mines, and ash from burned vegetation and soil.

PECO-relevant literature indicated that other land uses influence the amount of As pre-wildfire as well. Besides mining, urban/industrial and agricultural land-use are discussed in literature. Urban/industrial land use in the studies was associated with increased As concentration in surface water [48,49]. In Portugal, authors found increased post-wildfire As water concentrations near large agricultural sites [48]. As a result, the study suggested the potential for increased As concentrations from prior use of pesticides and fertilizers (such as phosphate containing fertilizers).

#### Building materials and urban environments as a media type impacted by wildfire.

The PECO-relevant literature included in this review generally did not address the influence of building materials such as Copper Chromated Arsenate (CCA)-treated lumber on wildfire mobilization and concentrations. However, there was supplementary literature that addressed the impact of CCA-treated lumber and wildfires burning into urban areas on ash concentrations [19,33]. Similar to other anthropogenic sources of As, concentrations from these sources of fuel were often higher than from natural sources unimpacted by industry and urban development [19,33]. In three articles, the ash concentration of As was elevated due to the combustion of buildings and building materials [18,19,33]. CCA-treated lumber specifically was discussed in one of the two studies measuring As concentration in ash samples [33]. It was found to have concentrations 33 times (CCA Sample: 807 mg/kg) that of other samples in the studies [33]. In two additional studies, As concentrations in ash samples near residential areas and near foundations of buildings ranged from 4.0 to 6.1 g/kg, with an elevated sample measuring 27 g/kg [19,33]. Finally, one supplemental article found the burning of anthropogenic materials by wildfires can be a source of As in smoke. In one sample in the study, there was a 2000% increase in As associated with PM_2.5_ on wildfire smoke impacted days vs non-smoke periods [31].

As noted in previous sections, the finding of higher As levels in wildfires burning the urban environment is consistent with the broader literature on metals and destructive fires. A suite of metals, including lead, copper, and nickel have been measured in air samples downwind of burning houses and vehicles [31]. Moreover, these metals have been found in ash following these wildfires (e.g., after the Marshall Fire in Colorado, USA), and in areas draining burned urban areas as well [32,50].

### Directionality of as concentration in water resources

#### Surface water.

Previous sections addressed common mobilization pathways and the influence of fuel type and impacted media. In this next section, the impact of wildfires and mobilization of As on water resources (both finished and unfinished) will be discussed.

The literature overwhelmingly indicates that wildfires increase fluxes of As in surface water. Arsenic concentrations increased or likely increased in 15 of the 17 PECO-relevant papers that examined surface water responses in surface water (S2 Table in Supplemental Tables). It is important to note that there was no average post-wildfire As concentration in surface water that eclipsed the 10 μg/L drinking water regulation across locations studied; yet, six of the papers reported instances of individually measured increases in As concentration post-wildfire over the 10 μg/L regulation, all of which occurred in untreated water. Of the seven papers that contained pre- and post-wildfire concentration values, all but one showed an increase (Fig 4). Rust et al. (2018) reported a 17% increase in the average concentration of As in surface water post-wildfire, whereas the remaining papers found increase in the maximum value ranging from 300% to 34,900%, the latter being down-gradient from the legacy mining operation (Fig 4).

The higher concentrations in the literature were largely in industrial impacted areas (primarily mining and agricultural land use). In the literature included, As concentrations were measured in surface water and sediment cores (which made it possible to assess the historical impacts of wildfires on surface water). The evidence suggests post-wildfire As mobilization into surface water most commonly occurs via runoff, and then secondary can occur through direct deposition and leaching. Runoff poses a prolonged risk to surface water as the destruction of natural barriers to runoff remains for months to years, allowing for each rain event to mobilize soil and ash containing As into nearby surface water. By contrast, direct deposition of ash and smoke, while increasing surface water As, has a shorter duration of effect, quickly declining once the wildfire is extinguished.

#### Groundwater.

Of the papers captured in the review, only three examined the impacts on groundwater. Among this limited literature, there were diverging results on the impacts to groundwater and private wells. In a paper that assessed wildfire impacts across the United States on groundwater, mean As concentrations increased 0.92 and 0.95 μg/L within a 3-year and 10-year window post-fire, respectively [5]. Notably, this represented an approximately 250% increase over pre-wildfire averages, and occurred in groundwater already treated for drinking water (Fig 4). In the same study, the violation rate (the number of times a public water system violates the As standard (10 μg/L) per year) increased by 0.32 in groundwater resources three years following wildfires (from 2.0 to 2.32) [5]. Although not statistically significant, increases in violation rates indicate that even small changes in As concentration on average can result in increases in As concentration that exceed drinking water safety standards.

In contrast, a groundwater study found no statistically significant relationship between time and As concentration fifteen days following a wildfire in Portugal [51]. Additionally, it did not find a difference in As concentration between unburnt and burnt areas [51]. The average As concentration across all sites ranged from 0.3 to 1.9 μg/L [51]. A final study assessing the impact directly on private wells also did not find detectable levels of As post-wildfire [52]. Although there were no pre-wildfire data available in this study, it can be assumed that, if there was any increase in As, it was insignificant and in such a low quantity that it was undetectable [52].

Overall, the impact of wildfires on As groundwater concentrations has been less studied compared to that of surface water concentrations. In the small number of studies published to date, the directionality of As in groundwater resources is inconsistent. The largest, most comprehensive study (e.g., Pennino et al. [5]) did find an impact, while a couple of more limited studies did not [51,52]. The limited and mixed outcomes of As concentration in groundwater and private wells suggest there needs to be additional research into As mobilization into groundwater post-wildfire.

### Overall literature gaps

There were some gaps in the literature that were either not covered in any of the studies included or there was limited coverage of the topic. Mobilization of As through landslides following wildfires was not covered by any of the literature. Like runoff and erosion—although to a potentially greater degree—landslides can move large amounts of sediment, which could mobilize As-containing minerals and compounds found in the soil following wildfires. There was also limited research on the impact of current fire mitigation and control strategies on As mobilization. Chemical fire retardants used to control wildfires may contain compounds that interact and impact the speciation of As in various media. Fire retardants that contain ammonia and phosphorus may result in speciation of As and enhance mobilization, although additional research is needed on this potential.

Finally, as noted previously, there are a limited number of studies and inconsistent findings on the wildfire-induced mobilization of As into groundwater and private wells. There were three studies that focused on the influences of wildfire on groundwater As concentration. However, the results were inconsistent between articles. Notably, it may be that changes in private drinking water wells are simply going undetected post-wildfire. Groundwater drinking water standards in the US were established by the Safe Drinking Water Act, but it does not regulate private drinking water wells [22]. Instead, testing and maintenance of private wells is noncompulsory in most states and is the responsibility of the landowner [10].

### Overall implications for human health

When considering the health effects of As or other chemicals, a key consideration is the length of exposure. Most As exposure-related health outcomes occur with chronic exposure over a lifetime. As wildfires activity increases, there is a potential for wildfires to become more frequent events in wildfire-prone areas of the US and globally. Wildfires could potentially lead to chronic exposures with the repeated mobilization of As present naturally from the soil or from contamination or anthropogenic materials. This is perhaps most likely in areas already with elevated arsenic, with the increase in wildfire activity generating more mobile forms of As (such as As(V) or As(III)), potentially leading to increased chronic exposures in these areas. The effects may be less likely in areas without naturally elevated As, yet still the possibility remains if human activities concentrate As on the landscape and it is subsequently mobilized by wildfire, such as was observed downgradient from legacy mining. In this latter case, As may become a chronic source of water contamination, and thus potentially increase exposure.

## Conclusion

Overall, this review synthesized information on the impacts to water resources from the mobilization of As by wildfires. The primary mobilization pathways found in the literature were runoff, direct deposition of ash and smoke, and leaching, with runoff by far the dominant pathway. Not surprisingly, pre-wildfire As concentrations influence post-wildfire As in water resources. Land-use, such as legacy mining or agriculture, can increase As on the landscape, leading to higher concentrations in water resources when subsequently mobilized by wildfire. Burning of material environments also resulted in higher concentration of As in waterbodies via runoff and deposition. Much more literature exists on surface water vs. groundwater impacts. In surface water, As concentrations almost always increased following wildfire. Individual samples did increase above the US As drinking water standard (10 μg/L) in select instances in untreated water, but on average stayed below this threshold. One study of groundwater treated for domestic consumption found As concentrations exceeded 10 μg/L in certain instances and caused an increase in drinking water violations [5]. Otherwise, the literature focused on groundwater was quite limited. Given the increase in wildfire activity in the western US and other locations globally, the mobilization of As and other contaminants are also likely to increase. The results of this study suggest As is likely to increase in surface water post-wildfire, for weeks to several years or more following the event, while the impacts on groundwater require further investigation.

## Supplementary Material

Supplement1 S1 Table. A total of 676 citations screened in the current study.
S2 Table. Data extracted from the 20 papers included for full review in the current study.
S3 Table. Study data used as input into Fig 3 within the main manuscript.
S4 Table. Study data used as input into Fig 4 within the main manuscript.
S1 Text. Search strings and specific search terms used to identify literature in this review.
S2 Text. List of literature identified as PECO-relevant.
S3 Text. List of literature identified as supplemental.
S1 File. PRISMA checklist.

## Figures and Tables

**Fig 1.**
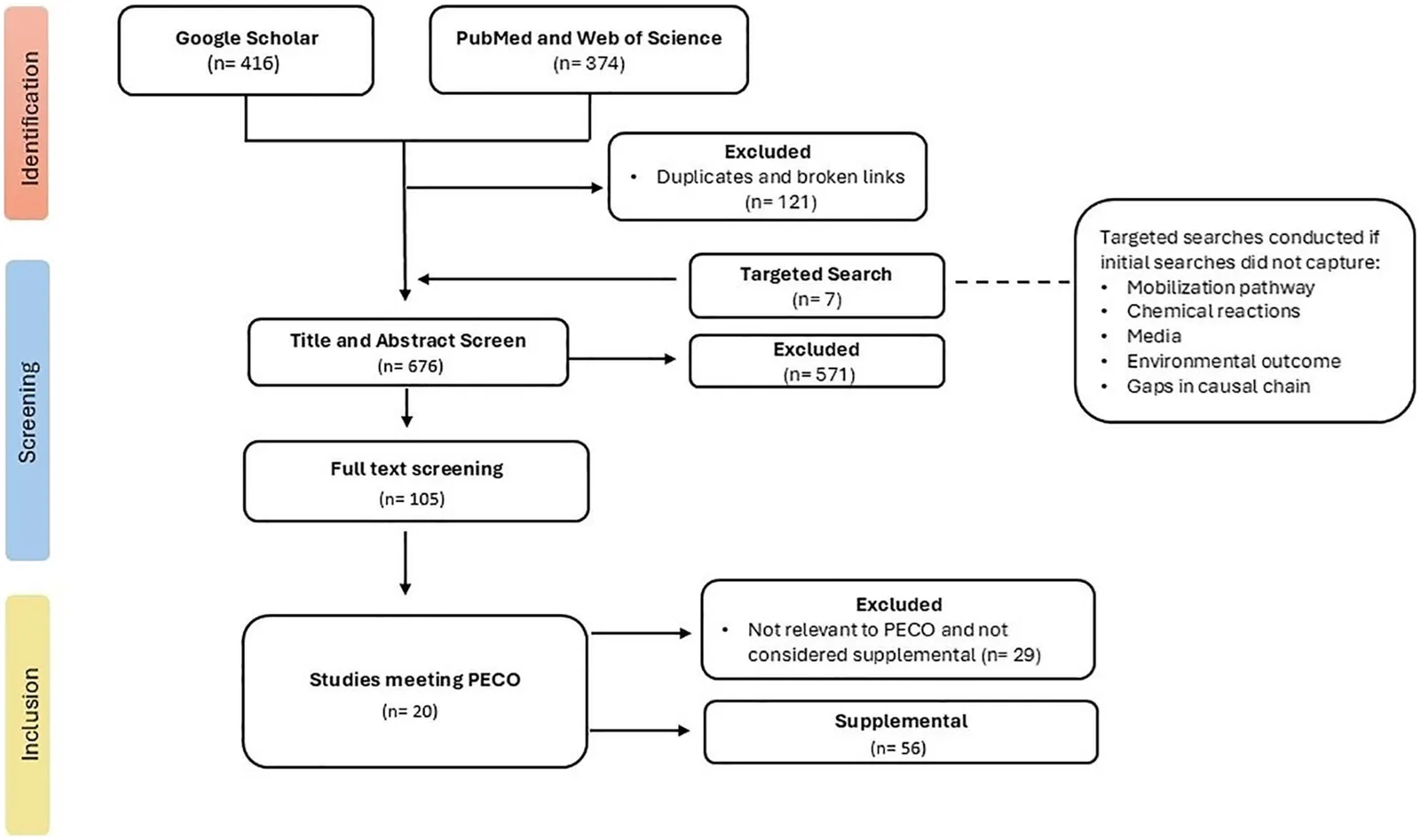
Prisma diagram of the literature search results. This diagram captures the review at each step and the amount of literature included or excluded at each part of the review.

**Fig 2.**
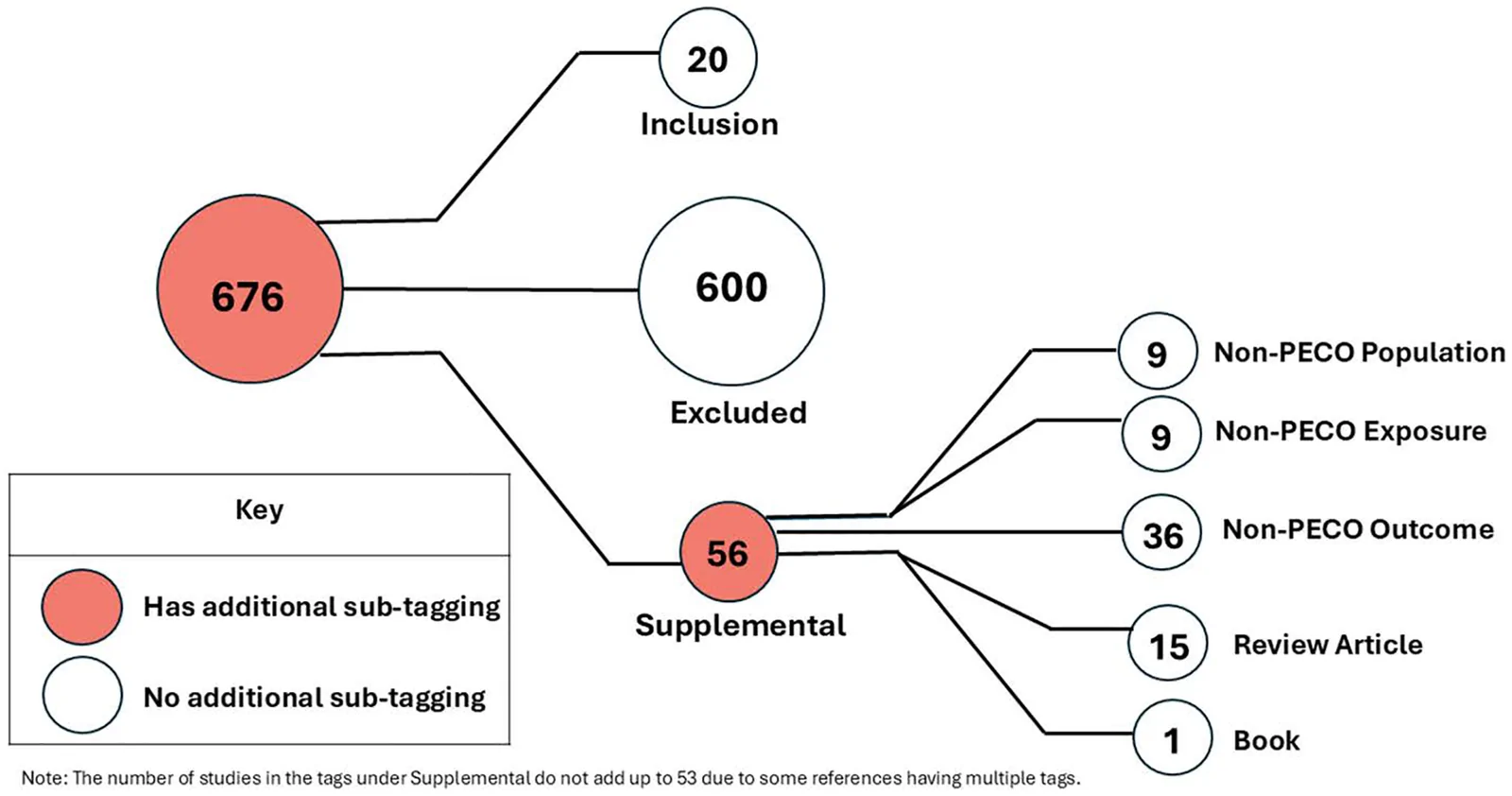
Literature tagging tree. The tagging tree represents the types of literature included and reasons for tagging as supplemental literature.

**Fig 3.**
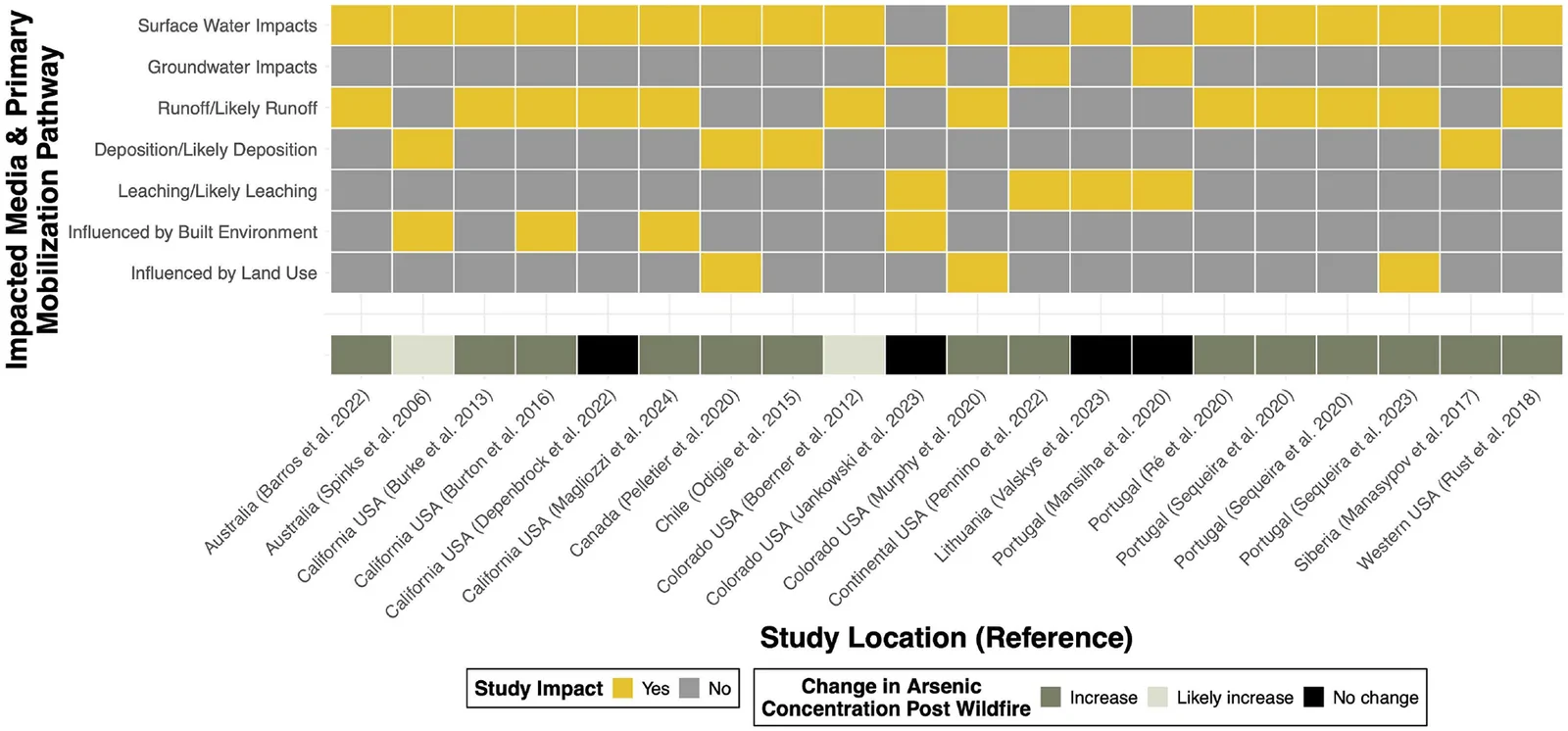
Water type, primary mobilization pathway, and modifying media impacted by wildfire. The heatmap illustrates the type of water (surface or ground), the primary mobilization pathway, and modifying media affected by wildfire according to the literature surveyed. The country or U.S. state where the wildfire occurred along with the reference are shown on the x axis, along with whether arsenic concentrations increased post-wildfire. An additional annotation row visualizes each study’s directionality in arsenic concentration post-fire. For specific data used as input for this figure, see S3 Table in Supplemental Materials.

**Fig 4.**
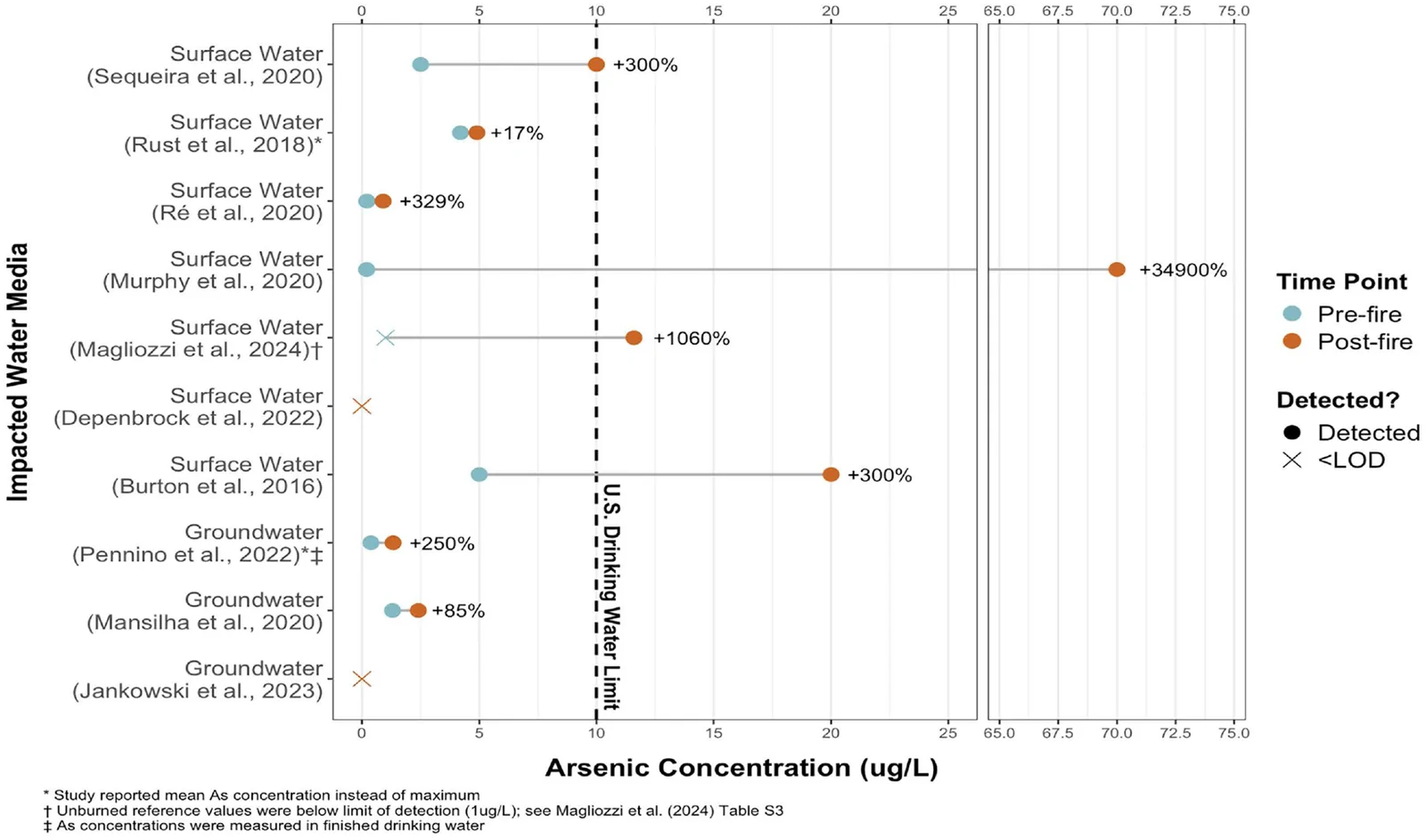
Change in arsenic concentrations pre- and post-wildfire. The forest plot shows pre- (blue) and post-wildfire (orange) arsenic (As) concentrations along with their percent change for articles reviewed containing this information. Shown are maximum values, except in two cases where means are shown as indicated. The dashed line represents the U.S. EPA’s drinking water limit of 10 μg/L. Values below the detection limit for individual studies are noted with a “X” rather than a dot. For specific data used as input for this figure, see S4 Table in Supplemental Materials.

**Table 1.** PECO criteria. The PECO Criteria were developed as part of this review to assess the relevance of literature for inclusion.

PECO criteria	This review’s definition of each criteria
**Population**	An area that experienced a wildfire
**Exposure**	Wildfire
**Comparator**	Impacted media (soil, vegetation, human-made structures, and building materials) and As concentrations prior to wildfires or other reference conditions
**Outcome**	Post-wildfire water resource (Groundwater and Surface Water) As concentration, either in water or sediments.

## Data Availability

All data can be found in the manuscript and supporting information files.
