# Supplementary material for: Wildfire mobilization of arsenic into water resources: A scoping review of current literature
Source: PLOS Water. Author manuscript; Available in PMC 2026 Oct 20. (PMC13679690; doi:10.1371/journal.pwat.0000549)
Supplement: Supplementary file 1 [file NIHMS2213732-supplement-Supplement1.zip › pwat.0000549.s005.docx]

**S1 Text. Search strings and specific search terms used to identify literature in this review.** The text below summarizes the search strings used to query each of the literature sources using in the current literature review. Search strings are provided for the primary source of literature, (**A**) Google Scholar, and the secondary sources of literature identified through Health and Environmental Research Online (HERO), (**B**) PubMed and (**C**) Web of Science.

**A. Google Scholar Search String**

(“Weather pattern”) OR (“firestorm”) OR (“Climate change”) OR (“fire retardant”) OR (“pyrogenic source”) OR (“Burned Area”) (“contaminated biomass”) OR (“biomass”) OR (“vegetation”) OR (“soil pollutant”) OR (“water pollutant*”) OR (“industrial waste”) OR (“biotransformation”) OR (“acid rain”) AND (“Wildfire”) OR (“prescribed fire”) AND (“Arsenic”) AND (“mobilization”) AND (“soil”) OR (“water*”) OR (“Surface water”) OR (“groundwater”) OR (“Air”) OR (“private well”) OR (“domestic well”) AND (“regionality”) OR (“contaminant flux”) OR (“soil erosion”) OR (“biogeochemical cycle”) OR (“chemical reaction*”) OR (“chromated copper arsenate”) OR (“industrial site”) OR (“Leaching”) OR (“hydrology”) OR (“combustion product") OR ("Smoke deposition")

**B. PubMed Search String**

PubMed (“Weather pattern*”[TW] OR “firestorm*”[TW] OR “Climate change*”[TW] OR “fire retardant*”[TW] OR “pyrogenic source”[TW] OR “Burned Area”[TW] OR “contaminated biomass”[TW] OR “biomass”[TW] OR “vegetation”[TW] OR “soil pollutant*”[TW] OR “water pollutant*”[TW] OR “industrial waste”[TW] OR “biotransformation”[TW] OR “acid rain”[TW]) AND (“fire*”[TW] OR “Wildfire*”[TW] OR “prescribed fire*”[TW]) AND “Arsenic”[TW] AND (“mobili*ation”[TW] OR “soil*”[TW] OR “water*”[TW] OR “Surface water*”[TW] OR “groundwater*”[TW] OR “Air”[TW] OR “private well*”[TW] OR “domestic well*”[TW] OR “regionality”[TW] OR “contaminant flux”[TW] OR “soil erosion”[TW] OR “biogeochemical cycle”[TW] OR “chemical reaction*”[TW] OR “chromated copper arsenate”[TW] OR “industrial site*”[TW] OR “Leaching”[TW] OR “hydrology”[TW] OR “combustion product*"[TW] OR "Smoke deposition"[TW])

**C. Web of Science Search String**

TS=(“Weather pattern*” OR “firestorm*” OR “Climate change*” OR “fire retardant*” OR “pyrogenic source” OR “Burned Area” OR “contaminated biomass” OR “biomass” OR “vegetation” OR “soil pollutant*” OR “water pollutant*” OR “industrial waste” OR “biotransformation” OR “acid rain”) AND TS=(“fire*” OR “Wildfire*” OR “prescribed fire*”) AND TS=“Arsenic” AND TS=(“mobili*ation” OR “soil*” OR “water*” OR “Surface water*” OR “groundwater*” OR “Air” OR “private well*” OR “domestic well*” OR “regionality” OR “contaminant flux” OR “soil erosion” OR “biogeochemical cycle” OR “chemical reaction*” OR “chromated copper arsenate” OR “industrial site*” OR “Leaching” OR “hydrology” OR “combustion product*" OR "Smoke deposition")
