# Supplementary material for: Wildfire mobilization of arsenic into water resources: A scoping review of current literature
Source: PLOS Water. Author manuscript; Available in PMC 2026 Oct 20. (PMC13679690; doi:10.1371/journal.pwat.0000549)
Supplement: Supplementary file 1 [file NIHMS2213732-supplement-Supplement1.zip › pwat.0000549.s006.docx]

**S2 Text. List of literature identified as PECO-relevant.** This list contains the Vancouver citations for literature from the primary (Google Scholar), secondary (HERO – Web of Science & PubMed), and targeted searches that were tagged as PECO-relevant. These citations are for articles included in the review in alphabetical order.

1. Barros TL, Bracewell SA, Mayer-Pinto M, Dafforn KA, Simpson SL, Farrell M, et al. Wildfires cause rapid changes to estuarine benthic habitat. Environmental Pollution. 2022 Sep;308:119571. **(GS)**
2. Boerner C, Coday B, Noble J, Roa P, Roux V, Rucker K, et al. Impacts of Wildfire in Clear Creek Watershed on the City of Golden’s Drinking Water Supply. **(GS)**
3. Burke MP, Hogue TS, Kinoshita AM, Barco J, Wessel C, Stein ED. Pre- and post-fire pollutant loads in an urban fringe watershed in Southern California. Environmental Monitoring and Assessment. 2013. 185: 10131-10145 (**GS**)
4. Burton CA, Hoefen TM, Plumlee GS, Baumberger KL, Backlin AR, Gallegos E, et al. Trace Elements in Stormflow, Ash, and Burned Soil following the 2009 Station Fire in Southern California. Khim JS, editor. PLoS ONE. 2016 May 4;11(5):e0153372. **(GS)**
5. Depenbrock S, Lane J, Asrat M, Poppenga R, Hargrave S, McNabb B, et al. Grazing lambs on pastures regrown after wildfires did not significantly alter metal content in meat and wool. Calif Agr. 2023 Feb;76(4):141–7. **(GS)**
6. Jankowski C, Isaacson K, Larsen M, Ley C, Cook M, Whelton AJ. Wildfire damage and contamination to private drinking water wells. AWWA Water Science. 2023 Jan;5(1):e1319. **(T)**
7. Magliozzi LJ, Matiasek SJ, Alpers CN, Korak JA, McKnight D, Foster AL, et al. Wildland–urban interface wildfire increases metal contributions to stormwater runoff in Paradise, California. Environ Sci: Processes Impacts. 2024;26(4):667–85. **(GS)**
8. Manasypov RM, Shirokova LS, Pokrovsky OS. Experimental modeling of thaw lake water evolution in discontinuous permafrost zone: Role of peat, lichen leaching and ground fire. Science of The Total Environment. 2017 Feb;580:245–57.
9. Mansilha C, Melo A, Martins ZE, Ferreira IMPLVO, Pereira AM, Espinha Marques J. Wildfire Effects on Groundwater Quality from Springs Connected to Small Public Supply Systems in a Peri-Urban Forest Area (Braga Region, NW Portugal). Water. 2020 Apr 17;12(4):1146. **(PM)**
10. Murphy SF, McCleskey RB, Martin DA, Holloway JM, Writer JH. Wildfire-driven changes in hydrology mobilize arsenic and metals from legacy mine waste. Science of The Total Environment. 2020 Nov;743:140635. **(PM & WOS)**
11. Odigie KO, Khanis E, Hibdon SA, Jana P, Araneda A, Urrutia R, et al. Remobilization of trace elements by forest fire in Patagonia, Chile. Reg Environ Change. 2016 Apr;16(4):1089–96. **(WOS)**
12. Pelletier N, Chételat J, Blarquez O, Vermaire JC. Paleolimnological Assessment of Wildfire‐Derived Atmospheric Deposition of Trace Metal(loid)s and Major Ions to Subarctic Lakes (Northwest Territories, Canada). JGR Biogeosciences. 2020 Aug;125(8):e2020JG005720. **(WOS)**
13. Pennino MJ, Leibowitz SG, Compton JE, Beyene MT, LeDuc SD. Wildfires can increase regulated nitrate, arsenic, and disinfection byproduct violations and concentrations in public drinking water supplies. Science of The Total Environment. 2022 Jan;804:149890. **(GS)**
14. Ré A, Campos I, Puga J, Keizer JJ, Gonçalves FJM, Pereira JL, et al. Feeding inhibition following in-situ and laboratory exposure as an indicator of ecotoxic impacts of wildfires in affected waterbodies. Aquatic Toxicology. 2020 Oct;227:105587.**(GS)**
15. Rust AJ, Hogue TS, Saxe S, McCray J. Post-fire water-quality response in the western United States. Int J Wildland Fire. 2018;27(3):203. **(GS)**
16. Sequeira MD, Castilho AM, Tavares AO, Dinis P. Assessment of superficial water quality of small catchment basins affected by Portuguese rural fires of 2017. Ecological Indicators. 2020 Apr;111:105961. **(WOS)**
17. Sequeira MD, Castilho AM, Dinis PA, Tavares AO. Impact Assessment and Geochemical Background Analysis of Surface Water Quality of Catchments Affected by the 2017 Portugal Wildfires. Water. 2020 Sep 30;12(10):2742.**(WOS)**
18. Sequeira MD, Castilho A, Tavares AO, Dinis P. The Rural Fires of 2017 and Their Influences on Water Quality: An Assessment of Causes and Effects. IJERPH. 2022 Dec 20;20(1):32. **(PM & WOS)**
19. Spinks J, Phillips S, Robinson P, Van Buynder P. Bushfires and tank rainwater quality: a cause for concern? J Water Health. 2006 Mar;4(1):21–8. **(PM)**
20. Valskys V, Kibirkštis G, Taminskas J, Ulevičius A, Ignatavičius G. Fires in Raised Bog: Their Influence and Changes to Geochemical Elements in Peat Layers. Land. 2023 Oct 20;12(10):1948. **(GS)**
