# Supplementary material for: Wildfire mobilization of arsenic into water resources: A scoping review of current literature
Source: PLOS Water. Author manuscript; Available in PMC 2026 Oct 20. (PMC13679690; doi:10.1371/journal.pwat.0000549)
Supplement: Supplementary file 1 [file NIHMS2213732-supplement-Supplement1.zip › pwat.0000549.s007.docx]

**S3 Text. List of literature identified as supplemental.** This list contains the Vancouver citations for literature from the primary (Google Scholar), secondary (HERO – Web of Science & PubMed), and targeted searches that were tagged as supplemental. These citations are for articles included in the review in alphabetical order. Literature tagged as supplemental either are review articles, meta-analysis articles, or meet part, but, not all PECO requirements.

1. Abraham J, Dowling K, Florentine S. The Unquantified Risk of Post-Fire Metal Concentration in Soil: a Review. Water Air Soil Pollut. 2017 May;228(5):175. **(WOS)**
2. Abraham J, Dowling K, Florentine S. Risk of post-fire metal mobilization into surface water resources: A review. Science of The Total Environment. 2017 Dec;599–600:1740–55. **(PM & WOS)**
3. Abraham J, Dowling K, Florentine S. Controlled burn and immediate mobilization of potentially toxic elements in soil, from a legacy mine site in Central Victoria, Australia. Science of The Total Environment. 2018 Mar;616–617:1022–34. **(PM & WOS)**
4. Abraham J, Dowling K, Florentine S. Influence of controlled burning on the mobility and temporal variations of potentially toxic metals (PTMs) in the soils of a legacy gold mine site in Central Victoria, Australia. Geoderma. 2018 Dec;331:1–14. **(WOS)**
5. Alam M, Alshehri T, Wang J, Singerling SA, Alpers CN, Baalousha M. Identification and quantification of Cr, Cu, and As incidental nanomaterials derived from CCA-treated wood in wildland-urban interface fire ashes. Journal of Hazardous Materials. 2023 Mar;445:130608. **(PM & WOS)**
6. Alexakis DE. Suburban areas in flames: Dispersion of potentially toxic elements from burned vegetation and buildings. Estimation of the associated ecological and human health risk. Environmental Research. 2020 Apr;183:109153.**(PM & WOS)**
7. Alexakis DE. Contaminated Land by Wildfire Effect on Ultramafic Soil and Associated Human Health and Ecological Risk. Land. 2020 Oct 27;9(11):409. **(GS)**
8. Alshehri T, Wang J, Singerling SA, Gigault J, Webster JP, Matiasek SJ, et al. Wildland-urban interface fire ashes as a major source of incidental nanomaterials. Journal of Hazardous Materials. 2023 Feb;443:130311. **(GS)**
9. Baker LL, Strawn DG, Rember WC, Sprenke KF. Metal content of charcoal in mining-impacted wetland sediments. Science of The Total Environment. 2011 Jan;409(3):588–94. **(PM & WOS)**
10. Belongia MF, Hammond Wagner C, Seipp KQ, Ajami NK. Building water resilience in the face of cascading wildfire risks. Sci Adv. 2023 Sep 15;9(37):eadf9534. **(GS)**
11. Betha R, Behera SN, Balasubramanian R. 2013 Southeast Asian Smoke Haze: Fractionation of Particulate-Bound Elements and Associated Health Risk. Environ Sci Technol. 2014 Apr 15;48(8):4327–35. **(PM & WOS)**
12. Bladon KD, Emelko MB, Silins U, Stone M. Wildfire and the Future of Water Supply. Environ Sci Technol. 2014 Aug 19;48(16):8936–43. **(GS)**
13. Boaggio K, LeDuc SD, Rice RB, Duffney PF, Foley KM, Holder AL, et al. Beyond Particulate Matter Mass: Heightened Levels of Lead and Other Pollutants Associated with Destructive Fire Events in California. Environ Sci Technol. 2022 Oct 18;56(20):14272–83. **(GS)**
14. Brucker CP, Livneh B, Minear JT, Rosario-Ortiz FL. A review of simulation experiment techniques used to analyze wildfire effects on water quality and supply. Environ Sci: Processes Impacts. 2022;24(8):1110–32. **(GS)**
15. Caumo S, Lázaro WL, Sobreira Oliveira E, Beringui K, Gioda A, Massone CG, et al. Human risk assessment of ash soil after 2020 wildfires in Pantanal biome (Brazil). Air Qual Atmos Health. 2022 Dec;15(12):2239–54. **(GS)**
16. Cesari D, Genga A, Ielpo P, Siciliano M, Mascolo G, Grasso FM, et al. Source apportionment of PM 2.5 in the harbour–industrial area of Brindisi (Italy): Identification and estimation of the contribution of in-port ship emissions. Science of The Total Environment. 2014 Nov;497–498:392–400. **(PM & WOS)**
17. Collar NM, Earles TA. Unique challenges posed by fire disturbance to water supply management and transfer agreements in a headwaters region. Journal of Environmental Management. 2023 Aug;339:117956. **(GS)**
18. Committee on the Chemistry of Urban Wildfires, Board on Chemical Sciences and Technology, Division on Earth and Life Studies, National Academies of Sciences, Engineering, and Medicine. The Chemistry of Fires at the Wildland-Urban Interface [Internet]. Washington, D.C.: National Academies Press; 2022 [cited 2025 Jun 11]. Available from: <https://www.nap.edu/catalog/26460> **(GS)**
19. Cooke SJ, Galassi DMP, Gillanders BM, Landsman SJ, Hammerschlag N, Gallagher AJ, et al. Consequences of “natural” disasters on aquatic life and habitats. Environ Rev. 2023 Mar 1;31(1):122–40. **(GS)**
20. Correa Velez KE, Alam M, Baalousha MA, Norman RS. Wildfire Ashes from the Wildland-Urban Interface Alter *Vibrio vulnificus* Growth and Gene Expression. Environ Sci Technol. 2024 May 14;58(19):8169–81. **(GS)**
21. El-Ghiaty MA, El-Kadi AOS. Arsenic: Various species with different effects on cytochrome P450 regulation in humans. EXCLI J. 2021;20:1184–242. **(GS)**
22. Esen A, Yushin N, Grozdov D, Yildiz C, Zinicovcaia I, Haciyakupoglu S, et al. Effect of wildfire on soil element concentrations in Mediterranean Türkiye. Journal of Radioanalytical and Nuclear Chemistry. 2023 Apr 18;322(11):4667–76. **(GS)**
23. Ferreira ARL, Sanches Fernandes LF, Cortes RMV, Pacheco FAL. Assessing anthropogenic impacts on riverine ecosystems using nested partial least squares regression. Science of The Total Environment. 2017 Apr;583:466–77. **(PM & WOS)**
24. Fernandez-Marcos ML. Potentially Toxic Substances and Associated Risks in Soils Affected by Wildfires: A Review. Toxics. 2022 Jan 11;10(1):31. **(GS)**
25. Green K. Migratory Bogong Moths (Agrotis Infusa) Transport Arsenic and Concentrate It to Lethal Effect by Estivating Gregariously in Alpine Regions of The Snowy Mountains of Australia. Arctic, Antarctic, and Alpine Research. 2008 Feb;40(1):74–80. **(WOS)**
26. Ivey C, Holmes H, Shi G, Balachandran S, Hu Y, Russell AG. Development of PM_2.5_ Source Profiles Using a Hybrid Chemical Transport-Receptor Modeling Approach. Environ Sci Technol. 2017 Dec 5;51(23):13788–96. **(PM & WOS)**
27. Johnston SG, Bennett WW, Burton ED, Hockmann K, Dawson N, Karimian N. Rapid arsenic(V)-reduction by fire in schwertmannite-rich soil enhances arsenic mobilisation. Geochimica et Cosmochimica Acta. 2018 Apr;227:1–18. **(GS)**
28. Johnston SG, Burton ED, Moon EM. Arsenic Mobilization Is Enhanced by Thermal Transformation of Schwertmannite. Environ Sci Technol. 2016 Aug 2;50(15):8010–9. **(PM & WOS)**
29. Johnston SG, Karimian N, Burton ED. Fire Promotes Arsenic Mobilization and Rapid Arsenic(III) Formation in Soil via Thermal Alteration of Arsenic-Bearing Iron Oxides. Front Earth Sci. 2019 May 29;7:139. **(GS)**
30. Kibria G, Nugegoda D, Rose G, Haroon AKY. Climate change impacts on pollutants mobilization and interactive effects of climate change and pollutants on toxicity and bioaccumulation of pollutants in estuarine and marine biota and linkage to seafood security. Marine Pollution Bulletin. 2021 Jun;167:112364. **(GS)**
31. Leach JA, Kelleher C, Kurylyk BL, Moore RD, Neilson BT. A primer on stream temperature processes. WIREs Water. 2023 Jul;10(4):e1643. **(GS)**
32. Lin YC, Hsu SC, Lin CY, Lin SH, Huang YT, Chang Y, et al. Enhancements of airborne particulate arsenic over the subtropical free troposphere: impact of southern Asian biomass burning. Atmos Chem Phys. 2018 Oct 1;18(19):13865–79. **(WOS)**
33. Liu Y, Ye X, Zhou B, Tian Z, Liu C, Li K. Potentially toxic elements in smoke particles and residual ashes by biomass combustion from Huangshi National Mine Park, China. Environ Geochem Health. 2023 Mar;45(3):629–45. **(GS)**
34. Lopez AM, Avila CCE, VanderRoest JP, Roth HK, Fendorf S, Borch T. Molecular insights and impacts of wildfire-induced soil chemical changes. Nat Rev Earth Environ. 2024 May 14;5(6):431–46. **(GS)**
35. Lynam MM, Dvonch JT, Barres JA, Morishita M, Legge A, Percy K. Oil sands development and its impact on atmospheric wet deposition of air pollutants to the Athabasca Oil Sands Region, Alberta, Canada. Environmental Pollution. 2015 Nov;206:469–78. **(GS)**
36. Megremi I, Stathopoulou E, Vorris E, Kostakis M, Karavoltsos S, Thomaidis N, et al. Mediterranean Wildfires’ Effect on Soil Quality and Properties: A Case from Northern Euboea, Greece. Land. 2024 Mar 3;13(3):325. **(GS)**
37. Miotliński K, Tshering K, Boyce MC, Blake D, Horwitz P. Simulated temperatures of forest fires affect water solubility in soil and litter. Ecological Indicators. 2023 Jun;150:110236. **(GS)**
38. Mir K, Rutter A, Koch I, Smith P, Reimer K, Poland J. Extraction and speciation of arsenic in plants grown on arsenic contaminated soils. Talanta. 2007 Jun 15;72(4):1507–18. **(PM & WOS)**
39. Moghaddam-Ghadimi S, Tam A, Khan UT, Gora SL. How might climate change impact water safety and boil water advisories in Canada? Joly Y, editor. FACETS. 2023 Jan 1;8:1–21. **(GS)**
40. Ocampos MS, Leite LCS, De Pádua Melo ES, De Cássia Avellaneda Guimarães R, Oliveira RJ, De Cássia Freitas K, et al. Indirect Methods to Determine the Risk of Damage to the Health of Firefighters and Children Due to Exposure to Smoke Emission from Burning Wood/Coal in a Controlled Environment. IJERPH. 2023 Apr 21;20(8):5607. **(GS)**
41. Pacheco FAL, Sanches Fernandes LF. Hydrology and stream water quality of fire-prone watersheds. Current Opinion in Environmental Science & Health. 2021 Jun;21:100243. **(GS)**
42. Paul MJ, LeDuc SD, Lassiter MG, Moorhead LC, Noyes PD, Leibowitz SG. Wildfire Induces Changes in Receiving Waters: A Review With Considerations for Water Quality Management. Water Resources Research. 2022 Sep;58(9):e2021WR030699. **(GS)**
43. Premović PI. Cretaceous-Paleogene Boundary Clays from Spainand New Zealand: Arsenic Anomalies. Open Geosciences. 2015 Nov 20;7(1):20150052. **(GS)**
44. Rao L, Zheng C, Chen JB, Cai JZ, Yang ZB, Xu XX, et al. Ecological and human health hazards of soil heavy metals after wildfire: A case study of Liangshan Yi autonomous prefecture, China. Chemosphere. 2024 Mar;352:141506. **(GS)**
45. Roberto OC, Martín Enrique JM, Ana Carolina RF, Joan Albert SC, Diana MF, Libia Hascibe PB. Historical fluxes of metal and metalloids in an aquatic ecosystem affected by land-use change and mining activities in northwestern Mexico. International Journal of Sediment Research. 2023 Oct;38(5):724–38. **(GS)**
46. Roshan A, Biswas A. Fire-induced geochemical changes in soil: Implication for the element cycling. Science of The Total Environment. 2023 Apr;868:161714. **(GS)**
47. Rothwell JJ, Evans MG, Allott TEH. In-Stream Processing of Sediment-Associated Metals in Peatland Fluvial Systems. Water Air Soil Pollut. 2007 Nov 29;187(1–4):53–64. **(GS)**
48. Sánchez-García C, Santín C, Neris J, Sigmund G, Otero XL, Manley J, et al. Chemical characteristics of wildfire ash across the globe and their environmental and socio-economic implications. Environment International. 2023 Aug;178:108065. **(GS)**
49. Saqib N, Bäckström M. Distribution and leaching characteristics of trace elements in ashes as a function of different waste fuels and incineration technologies. Journal of Environmental Sciences. 2015 Oct;36:9–21. **(PM & WOS)**
50. Sardans J, Peñuelas J, Estiarte M. Warming and drought change trace element bioaccumulation patterns in a Mediterranean shrubland. Chemosphere. 2008 Jan;70(5):874–85. **(GS)**
51. Sutton OF, McCarter CPR, Waddington JM. Globally-significant arsenic release by wildfires in a mining-impacted boreal landscape. Environ Res Lett. 2024 Jun 1;19(6):064024.**(GS)**
52. Terzano R, Rascio I, Allegretta I, Porfido C, Spagnuolo M, Khanghahi MY, et al. Fire effects on the distribution and bioavailability of potentially toxic elements (PTEs) in agricultural soils. Chemosphere. 2021 Oct;281:130752. **(GS)**
53. Thomson B, Rose M. Environmental contaminants in foods and feeds in the light of climate change: Climate change and environmental contaminants in food. Quality Assurance and Safety of Crops & Foods. 2011 Mar;3(1):2–11. **(WOS)**
54. Tuhý M, Ettler V, Rohovec J, Matoušková Š, Mihaljevič M, Kříbek B, et al. Metal(loid)s remobilization and mineralogical transformations in smelter-polluted savanna soils under simulated wildfire conditions. Journal of Environmental Management. 2021 Sep;293:112899. **(PM & WOS)**
55. Wan X, Li C, Parikh SJ. Chemical composition of soil-associated ash from the southern California Thomas Fire and its potential inhalation risks to farmworkers. Journal of Environmental Management. 2021 Jan;278:111570. **(GS)**
56. Zhou C, Liu G, Wang X, Qi C, Hu Y. Combustion characteristics and arsenic retention during co-combustion of agricultural biomass and bituminous coal. Bioresource Technology. 2016 Aug;214:218–24. **(PM & WOS)**
